# 

**Published:** 1889-02

**Authors:** 


### TABLE OF CONTENTS.

ORIGINAL AND SELECTED.

Calomel: its properties, &c., by H. Christopher, M. D. .... 41
Retrocedent Measles by A. M. Lawson, M. D, 48
Laxatives in Childbed, by E. Van Goidtsnoven, M. D. .... 49
The History of Ang. Pect. by R. L. Bowcock, M. D. .... 50
Tapping for Acites, by B. W. Richardson, M. D. 51
Alkaloidal Therapeutics, by C. D. Wescott, M. D. .... 52
Salix Nigra, &c. by F. T. Paine. M. D. .... 54

ABSTRACTS &c.

Pneumonia .... 56
Dr. I. M. Shaw on Chorea .... 57
Med. Treat. Morphine Habit .... 58
Precautions as to Hypod Medication; The combination of Sod. Chlor. and Cor. Sub. .... 59
Use of Pilocarp in Mid. Ear Diseases .... 60
Possible Sub. for Tracheotomy &c. .... 61
Nasal origin of Whoop. Cough, &c ; Opium .... 62
Treat. of Anal Fissure; Eruptions due to Antipyrine; Thuya Oc. in Epithel. of Larynx; .... 63
Strychnia an antidote; Salix Niger; Warts .... 64
Pleasant Purgative for children; Some advantages of Memb. in Med. So. .... 65
Felons; Albuminaria in pregnancy .... 66
Treat. of Incon. of Urine, &c. .... 67
Method of destroying Tattoo Marks .... 68
Cin. Med. News com. on death of a physician; Dysmenorrhœa .... 69
Antipyrine as an Anodyne in Labor .... 70

SCIENTIFIC

No need of weather bureaus; An electro Magnet; Dr. Juneman; The artesian well; Oersted's discovery .... 71
Novel scheme for harbor defence; Elastic Traces .... 72

NOTES AND FORMULÆ.

Laxative in childbed; Prairie Itch; Eczema of Head; Incont. of Urine; Earache .... 73
Palpitation; Treat. of Warts by Arsenic; Treat. of Warts; Em. in Skin Diseases; Cough Remedy .... 74

EDITORIAL.

Charging ex Doctors; The human body .... 75
Enlarged Spleen; The Doctor at home .... 76
Advertisers, &c. .... 77, 78, 79
Special Notes .... 79, 80

